# Is It a Fraud?

**Published:** 1890-03

**Authors:** 


### "Is it a Fraud?"

A preparation has been offered and sold, quite extensively, through many parts of the United States during the last three or four years, as a local anæsthetic to be used mainly in the extraction of teeth. It is known as "Steinau's Local Anæsthetic, for the Painless Extraction of Teeth." The claim is that its composition is known only to the person whose name it bears, and in this he claims a proprietorship, and demands a license fee of $25 to $50 of those whom he can induce to use it and pay the sum demanded. A bottle of this material just purchased of one of his agents, was a short time ago put into the hands of the editor for examination, and having a desire to know beyond question as to the composition of this material, and desiring additional evidence to his own analysis, it was placed in the hands of Mr. F. G. Novy, M. S. Instructor of Hygiene and Physiological Chemistry in the University of Michigan, and after a careful analysis his report is that the solution contains three-quarters of one per cent. of cocaine, with a very small amount of carbolic acid—but little more than a trace—this added probably to prevent change of the cocaine, or with that view at least, it might have been used for a purpose far less commendable than this. Not a trace of any other substance was found in the mixture.

The acting agent in this preparation is cocaine and nothing else. Now we trust that this statement will answer the query contained in the caption of this article; and let us all hope and work for the hastening of the day when all frauds, shams and impositions shall be swept out as with the besom of destruction, and their authors and promotors either be converted or brought to shame and confusion.

